# A Modern Approach to Endometrial Carcinoma: Will Molecular Classification Improve Precision Medicine in the Future?

**DOI:** 10.3390/cancers12092577

**Published:** 2020-09-10

**Authors:** Simone Marnitz, Till Walter, Birgid Schömig-Markiefka, Tobias Engler, Stefan Kommoss, Sara Yvonne Brucker

**Affiliations:** 1 Department of Radiation Oncology, CyberKnife and Radiotherapy, Medical Faculty University Cologne, 50937 Cologne, Germany; till.waltar@uk-koeln.de; 2 Center of Integrated Oncology Cologne Bonn (CIO), 53127 Bonn, Germany; birgid.markiefka@uk-koeln.de; 3 Institute of Pathology, Medical Faculty University Cologne, 50937 Cologne, Germany; 4 Department of Women’s health, University Hospital Tuebingen, 72076 Tuebingen, Germany; tobias.engler@med.uni-tuebingen.de (T.E.); stefan.kommoss@med.uni-tuebingen.de (S.K.)

**Keywords:** endometrial cancer, uterus carcinoma, molecular classification, risk classification, risk stratification, adjuvant radiation, brachytherapy, POLE, L1CAM, MMRd

## Abstract

**Simple Summary:**

The scientific community widely agrees that molecular classification will be key to endometrial carcinoma therapeutic strategies in the future. Retrospective analyses of large endometrial carcinoma patient cohorts gave rise to a new understanding of one of the most relevant gynecologic malignancies. Potentially replacing the current type I and type II terminology, four molecular subtypes have been established, each of them reflecting underlying molecular aberrations and distinct clinical behavior. Future research will have to focus on how to integrate these new findings into clinical practice with the ultimate goal to drive personalized endometrial carcinoma patient care forward.

**Abstract:**

Endometrial cancer has been histologically classified as either an estrogen-dependent cancer with a favorable outcome or an estrogen-independent cancer with a worse prognosis. These parameters, along with the clinical attributions, have been the basis for risk stratification. Recent molecular and histopathological findings have suggested a more complex approach to risk stratification. Findings from the Cancer Genome Atlas Research Network established four distinctive genomic groups: ultramutated, hypermutated, copy-number low and copy-number high prognostic subtypes. Subsequently, more molecular and histopathologic classifiers were evaluated for their prognostic and predictive value. The impact of molecular classification is evident and will be recognized by the upcoming WHO classification. Further research is needed to give rise to a new era of molecular-based endometrial carcinoma patient care.

## 1. Introduction

For many decades, endometrial cancer has been considered a tumor entity with a favorable outcome and two distinct types: Type 1, estrogen-dependent with a better prognosis, and Type 2, non-estrogen-dependent with a worse prognosis [1]. Endometrial carcinomas (ECs) are typically diagnosed at an early stage and have more favorable outcomes; however, between 15–20% of women will relapse and eventually die from their disease. Identifying which patients are at risk for recurrence may be challenging; consequently, under- and overtreatment remain clinical issues. Endometrial carcinoma is the most common gynecological malignancy, and progress in accurate and reliable categorization is eagerly awaited.

### 1.1. Current Risk Stratification

**Figure 1:**
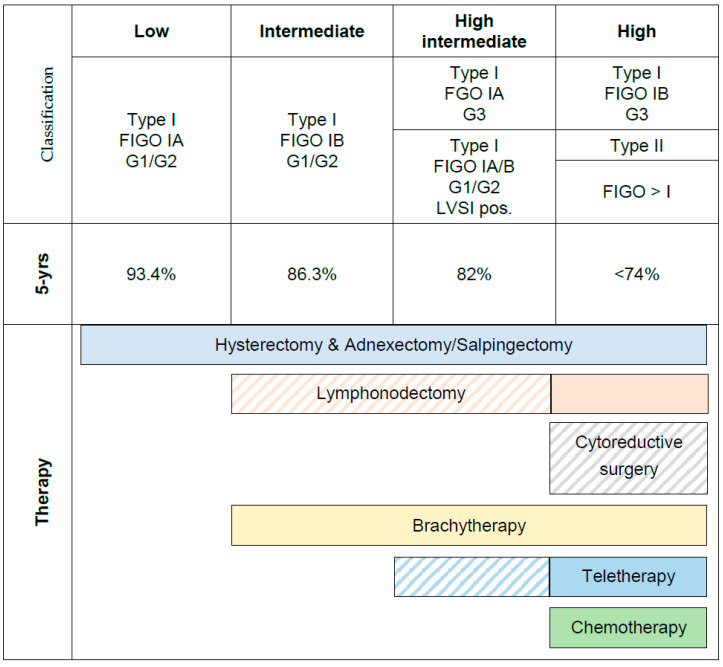
Current recommendations on risk stratification and therapy based on classical clinical risk factors and five-year overall survival (modified from AGO S3-Guideline).

Based on tumor type, stage, grade and lymphovascular space invasion (LVSI) current endometrial carcinoma treatment guidelines (ESMO-ESGO-ESTRO 2016 Consensus) stratify patients into four distinctive groups: low-, intermediate-, high-intermediate- and high-risk disease [2,3,4,5]. Treatment decisions and patient guidance have been tailored to the latter risk groups. However, comparison of treatment recommendations may be difficult since the international scientific community has used different risk classifications in the past and has not yet been able to implement a standardized classification system [6,7,8,9,10] (Figure 1).

### 1.2. Limitations of Clinical Risk Factors

Furthermore, indication for more extensive surgery besides hysterectomy with bilateral salpingo-oophorectomy (BSO) depends on factors that are often unknown or unclear preoperatively (i.e., myometrial infiltration). These preoperative uncertainties lead to either undertreatment with subsequent second surgery or the risk of overtreatment. Maximum tumor size and myometrial infiltration can be determined using intraoperative frozen sections, which are not available everywhere [9,11]. Of course, if future treatment also depends on increasingly complex analyses like molecular classification, there is a need for centralization of the diagnostic procedure and even the gynecological cancer treatment. The German Cancer Aid and the National Cancer Plan are working together with cancer societies with the overall goal to centralize cancer patient treatment and to provide care to a reasonable number of patients per year with a multidisciplinary team of specialists. This is a “conditio sine qua non”.

Moreover, the validity of clinical risk factors is often limited by a large interobserver variability in conventional pathology with regard to LVSI and grading, which could also be different from the result of the biopsy taken by curettage to the uterus specimen. The re-evaluation by a central pathology, compared to the results of a local pathology, demonstrated a considerable shift in grading EC patients that impacts the reliability of an adjuvant treatment recommendation [10,12].

Lymph node metastases (pN+) are considered a major risk factor and are the most important prognostic factor and determinator for adjuvant therapy in patients with EC.Moreover, most primary ECs are confined to the uterus, and only about 10% will have pelvic lymph node metastasis [13].However, as a result of the ASTEC and Italian trial [14,15], there has been a decreasing rate of lymphadenectomies (LNs) worldwide. As a consequence, FIGO stage III cannot be subdivided into stage III C1 and C2 in many patients, which makes the interpretation of study results difficult (please see the section below) [16,17,18].

Although not validated in randomized studies, there is an increasing use of the sentinel node technique (SLN) in endometrial cancer patients, taking into account that the SLN minimizes radicality but could also maximize the accuracy of the staging and tumor stage, as up to 20% of positive SLNs are atypically located and could therefore be missed by conventional LNs. Immunohistochemistry in combination with ultrastaging of sentinel lymph nodes leads to a dramatic increase in detection of isolated tumor cells (ITCs) and micrometastases (MICs). For adjuvant treatment recommendations, the SLN raises new questions. ITCs and MICs now account for 50% of the positive findings after ultrastaging in EC patients [19]. The impact of ITCs on prognosis is a topic of ongoing discussion. MICs lead to a significantly worse prognosis and are considered as pN+ disease (lymph node metastases) and therefore lead to an increased rate of adjuvant treatment as a result of SLN [19,20,21,22,23]. These results were underlined by the FIRES trial. It was demonstrated that 54% of the patients with positive sentinel lymph nodes had lymph node disease identified only with ultra-staging. This results in >50% of adjuvant treatment indications compared to cohorts only after conventional histology. Sixty percent of the FIRES patients with positive SLN had disease limited to the sentinel lymph nodes. Seventeen percent of the patients had positive sentinel lymph nodes that were found exclusively in regions lying outside of a routine lymphadenectomy. This should lead to rethinking a target volume definition for radiation [24]. However, despite more frequent use of adjuvant therapy following sentinel concept, a survival benefit has not yet been demonstrated until now [19,20,21,22,23].

Despite the supposedly differentiated clinical risk factors, the clinical impression remains that some patients classified as low-risk have an unfavorable course of the disease, while some patients with high risk factors show an impressively long progression-free survival. Until now, it has not been possible to predict such unexpected behavior; therefore, frequent follow-up visits are recommended for all patients, and under- and overtreatment remain daily routine practice obstacles. This solidified the impression that the usual clinical risk classification does not adequately depict tumor biology [25]. Furthermore, histotype and grade, the key parameters in the most popular stratification systems, have been shown to have a poor reproducibility [26,27,28,29,30], resulting in clinically relevant discrepancies. In terms of assessing treatment efficacy and refining these histopathological prognosticators, the low interobserver agreement has hampered research for many years. Moreover, stage and lymphovascular space invasion (LVSI), which are currently crucial for risk stratification, are only available after definitive surgery, leading to risk models that are unable to aid in decision making with regards to the extent of surgery. In contrast to more uniform treatment approaches of other gynecological malignancies, endometrial carcinoma management varies widely across international guidelines currently in use. 

Therefore, new predictive tissue markers are warranted. Those markers could either be used alone or in combination with traditional clinico-pathological diagnostics to develop a preoperative nomogram to improve diagnostic accuracy and guide clinicians in their treatment decisions. This will minimize explorative lymph node staging surgery and clinically guide informed surgical treatment.

The need to address the above-mentioned challenges was recognized by the international research community, and remarkable progress has recently been made. Current literature provides new insights not only into tumor biology, but also into clinical risk stratification. Two major findings have changed the landscape of how we approach endometrial carcinoma today. In 2013, the Cancer Genome Atlas (TCGA) accomplished the most comprehensive molecular study yet in endometrial carcinoma, stratifying the entity in four distinctive subgroups: The “ultramutated” subgroup is characterized by a strong association with mutations in the exonuclease domain of polymerase-ε (POLE) and also by an excellent prognosis. In addition to this intriguing subgroup, molecular profiling also identified a subgroup in which microsatellite instability (MSI) status was identified as a discriminating feature; a “copy-number high” subgroup with p53 mutations and a generally unfavorable course of disease and finally, the “copy-number low” subgroup. It has been suggested, that these molecular features may be used not only for patient classification and risk stratification but may also give rise to a more individualized guidance of surgery, adjuvant strategies and patient observation. However, the methods applied by TCGA were not feasible in a clinical context or single-patient setting. Inspired by these findings, molecular classifiers that are simplified, straightforward and easily applicable were developed, one of which is termed “ProMisE” (Proactive Molecular Risk Classifier for Endometrial Cancer) [31]. ProMisE identifies four molecular subtypes that are analogous but not identical to the four genomic subtypes described in TCGA. After development and confirmation, the classifier was validated in a large population-based series. The tool can be applied to standard formalin-fixed paraffin-embedded tissue using either endometrial biopsy or hysterectomy samples [32]. A similar assay has been established by the TransPORTEC international consortium, identifying very similar molecular subtypes with distinct outcomes and high diagnostic reproducibility [33,34]. 

At the same time when TCGA classification was published, the L1 neuronal cell-adhesion molecule (L1CAM) gained attention as a specific prognosticator and potential therapeutic target in early-stage type I endometrial carcinoma [35,36]. Multiple studies have shown the prognostic significance of L1CAM immunohistochemistry (IHC) in large cohorts of endometrial carcinoma [37,38,39,40]. According to the findings, in low-risk endometrial carcinoma, it has been suggested to limit the term “low-risk endometrial carcinoma” to L1CAM-negative tumors [40]. While L1CAM protein expression has been connected to serous and clear cell histology as well as abnormal p53 status (subsequently correlating with worse outcome), there is also evidence suggesting a p53-independent L1CAM in endometrial carcinoma molecular subtypes [35,41,42,43]. Thus, L1CAM expression may help further stratify risk after molecular classification. However, while L1CAM-positive tumors clearly have worse outcomes, it is prudent to maintain the original TCGA tumor subgroups and to not consider L1CAM tumors a subtype but rather as a prognostic subgroup of the large p53 wild-type group with no specific molecular risk profile (NSMP) and perhaps of MMR-D tumors [44]. In summary, molecular classification as well as L1CAM immunohistochemistry will play a key role in future algorithms to tailor adjuvant treatment and patient follow-up strategies. 

Future treatment strategies may include surgical considerations such as lymphonodectomy in low-risk endometrioid, FIGO stage Ia and G1/2 tumors if an integrated risk assessment indicates a rather unfavorable prognosis. Likewise, a modern integrated risk assessment will eventually help to identify patients in which more radical procedures such as systematic lymphonodectomy, omentectomy or multivisceral debulking attempts could contribute to longer progression-free survival (PFS) and overall survival times. Evaluation of adjuvant treatment modalities may be tailored to specific molecular features such as poly ADP ribose polymerase (PARP) inhibitors in p53 abnormal tumors, immune checkpoint inhibition or (chemo-)radiation in patients with a mismatch repair deficient (MMRd) subtype. Finally, patient follow-up might follow a less strict schedule and more cost-effective postoperative wait-and-see strategy if a bona fide low-risk situation is molecularly confirmed. In order to allow optimal upfront patient counseling, molecular profiling should be available at the time of endometrial biopsy. 

### 1.3. Current Recommendations for Adjuvant Treatment in Low-Risk Disease and Intermediate-Risk Parameters

Guideline recommendations should be evidence-based. The comparability of the results from randomized trials in EC is limited, since different definitions of prognostic subgroups are used. The actual guidelines recommend no adjuvant treatment in low-risk disease and brachytherapy in patients with stage I and intermediate-risk parameters (see Figure 1). These guidelines are based on the classical risk factors according to Figure 1.

#### 1.3.1. Radiation Needed in Stage I?

First-generation studies on adjuvant treatment demonstrated that, in stage I patients, external beam radiation decreases the pelvic and the vaginal recurrence rate significantly, but without impact on overall survival [45,46]. What always gets lost in the discussion about the value of stage I radiation therapy is the fact that overall survival was not the end point of the study. However, patients with grade 3 tumors and >50% myometrial invasion achieved an improved survival rate after external body radiation therapy (EBRT) [4,46,47,48]. Subgroups of patients with conventionally defined high-intermediate risk parameters benefited in terms of survival. However, this was a strong indicator that patient selection for the adjuvant studies was problematic, based on what was known at the time. This kept the discussion alive, in which patients could benefit from more aggressive adjuvant therapy that could lead to the evaluation of chemotherapy for adjuvant treatment [5,25]. 

#### 1.3.2. Radiation, Chemotherapy or Both: Who Could Profit?

The second generation of adjuvant treatment studies in EC evaluated whether chemotherapy or radiation is superior. Three randomized trials evaluated the use of chemotherapy versus radiation in patients with intermediate- or high-risk disease, and two of them failed to show any benefit from chemotherapy over radiotherapy [49,50]. The randomized trial by Randall et al. (GOG-122) is the most cited and misunderstood study in this context [51]. In the study, researchers used doxorubicin 60 mg/m^2^ and cisplatin 50 mg/m^2^ every three weeks for seven cycles, followed by one cycle of cisplatin in women with FIGO stage III or IV endometrial carcinoma with a maximum of 2 cm of residual disease after surgery. This demonstrated better PFS when compared with patients who underwent a (not indicated) whole abdominal irradiation with insufficient doses [52]. Results should be interpreted with caution, since patients with palliative situations were treated with insufficient radiation doses versus chemotherapy combination with unacceptably high rates of late neurologic toxicity.

The third-generation studies considered that patients with clinical risk factors have both an increased risk of local recurrence and an increased risk of distant metastases. This study evaluated the simultaneous and sequential chemotherapy + radiation versus radiation. 

The first third-generation study, the PORTEC-3 intergroup trial, investigated the potential benefit of concomitant chemoradiation followed by adjuvant chemotherapy versus pelvic radiotherapy alone for women with high risk for recurrence (FIGO stage I grade 3 with deep myometrial invasion LVSI, or both; stage II or III; or serous and clear cell histology). Six hundred and eighty-six women were randomly allocated to radiation (RT) with 48.6 Gy or chemoradiation (two cycles of concomitant cisplatin 50 mg/m^2^ in week one and week four of radiation, followed by four cycles of carboplatin AUC5 and paclitaxel 175 mg/m^2^ at three-week intervals (CRT-Ch)). Primary endpoints were overall survival (OS) and failure-free survival (FFS). After a median follow-up time of five years, five-year OS for CRT-Ch vs. RT was 81.8% versus 76.7%, respectively (*n.s.*). Patients with stage III EC, which included IIIA, IIIB and an unknown correct number of stage IIIC1/2 had the greatest benefit from the combined treatment: five-year FFS for stage III was 69.3% for CRT-Ch versus 58.0% for RT (*p* = 0.032). In addition, patients >70 years with FIGO stage III, serous histology or both had a greater benefit in subgroup analyses [18,53].

In contrast to PORTEC3, the GOG-258/NRG oncology study tested adjuvant chemotherapy alone versus chemoradiation. Patients with “optimally” debulked EC (<2 cm residual tumor) were tested. Pelvic ± paraaortic “lymph node sampling” was optional. Stage III/IVA endometrioid and FIGO stage I/II serous cancers were eligible and were assigned to either chemoradiation (EBRT *n* = 174 or EBRT ± brachytherapy with concomitant cisplatin 50 mg/m^2^ d1, d29, *n* = 204) followed by carboplatin AUC5 plus paclitaxel 175 mg/m^2^ q21 × 4 (CRT-Ch) versus chemotherapy only (Ch; carboplatin AUC6 and paclitaxel 175 mg/m^2^ q21 × 6) [54].

Chemoradiation halved the rates of pelvic and vaginal recurrences compared to chemotherapy (19% versus 10% and 7% versus 3%, respectively). Nevertheless, no difference in five-year OS was demonstrated. After five years, 21% and 27% of the patients after chemo and chemoradiation developed distant metastases [55].

The third, more recently published trial [56] was GOG-0249. This was an open-label phase III trial on the impact of recurrence-free survival (RFS) of EBRT (1.8/2.0 for 25–28 fractions) versus vaginal brachytherapy in combination with chemotherapy (paclitaxel at 175 mg/m^2^ followed by carboplatin AUC 6 over 45 min), repeated every 21 days for three cycles (VCB/C) in women in FIGO stage I and high-intermediate risk criteria, stage II disease, or stage I to II serous or clear cell tumors. Consistent with the above-mentioned results of the GOG-258/NRG oncology study, EBRT reduced the rate of pelvic and paraaortic recurrences by 50% compared to chemotherapy and brachytherapy. As expected, vaginal recurrences were low (2.5%) in both arms. Surprisingly, 18% of the patients developed distant metastases in both arms within five years. 

#### 1.3.3. The Need for Additional Differentiation of EC to Predict Adjuvant Therapy

In sum, recently published studies that should have provided clear evidence for the differentiated use of adjuvant therapies disappointed researchers with their partially contradictory results. The key to understanding the disappointing results is the inappropriate selection of patients based on clinical parameters. All included studies had a mixture of subgroups and cohorts that were biologically different. Patients benefited from the therapy to varying degrees; therefore, no consequences were clearly derived from the studies.

### 1.4. Response Predicition for Chemotherapy and Radiation

In addition to the prognostic value of genetic profiles, the question as to whether the genomic profile might have a predictive value for any adjuvant treatment is of great interest. Recently, Reijnen et al. were able to demonstrate MMRd molecular status as a predictive marker for response to adjuvant radiotherapy [57]. The authors were able to retrospectively investigate the predictive value of ProMisE MMR status within a large multicenter patient cohort of high-risk, stage IB or II grade 3 endometrioid endometrial carcinoma with and without adjuvant radiotherapy. Improved survival benefit was limited to patients with MMR-deficient tumor EECs (hazard ratio 0.19, 95% CI 0.05–0.77), and thus MMR status may contribute to personalized patient selection criteria in a high-risk scenario [57,58].

The radiosensitivity index (RSI) is a previously validated multigene expression index that estimates tumor radiosensitivity. Mohammadi et al. [59] validated the index in 204 patients with EC, of which 83 (41%) were treated with adjuvant RT. The median follow-up was 38.5 months. In patients treated with radiation, radioresistant tumors showed a worsening three-year pelvic control (84% vs. 100%; *p* = 0.02) with a worsening progression-free survival (three-year PFFS 65% vs. 89%; *p* = 0.04). More data are needed to derive a clinical implication.

### 1.5. New Study Landscape

The PORTEC 4a Study, which is currently recruiting patients, is the first clinical trial applying molecular-integrated risk profiles in primary endometrial carcinoma patients. The PORTEC 4a study is a randomized trial of molecular-profile-based versus standard recommendations for adjuvant radiotherapy in women with early-stage endometrial cancer. The primary objective of this study is to establish the rates of vaginal relapse in patients with high-intermediate-risk endometrial carcinoma treated with vaginal brachytherapy based on clinicopathological (standard) indications and compare these rates with those of patients who have receive molecular risk profile based recommendations for vaginal brachytherapy, observation or external beam radiotherapy. While patients are treated with vaginal brachytherapy in the standard arm, treatment decisions are based on an integrated molecular risk classification in the experimental arm (2:1 randomization). Patients will either be observed after surgery and followed closely for vaginal recurrence (low molecular risk profile, POLE mutation or absence of MMRd and CTNNB1 exon 3 wild-type) or they will receive vaginal brachytherapy as mentioned above (intermediate molecular risk profile). If molecular risk assessment indicates a high-risk situation, patients are recommended external beam radiation instead of vaginal brachytherapy (p53 abnormal or L1CAM positivity or substantial lymphovascular space invasion [60]).

### 1.6. New Guidelines for Adjuvant Treatment

Due to the convincing data, the ESGO guideline committee decided to adapt the European guidelines on adjuvant treatment in endometrial cancer on the basis of new risk profiles (Table 1). The new guidelines will be published soon. A European proposal on new recommendations for adjuvant treatment is under discussion and will be published this year (Table 2).

## 2. Conclusions

The new insights into genomic classification open up new horizons for a better understanding of the biology of endometrial cancer, differentiated risk stratification, improved estimation of the prognosis and therapy response, de-escalation of adjuvant therapies for patients who do not benefit from them and escalation of adjuvant measures for patients with an aggressive illness. Recently, our group investigated if TCGA-inspired endometrial cancer molecular subtyping also stratifies the prognosis for a specific type of ovarian cancer (the endometrioid ovarian carcinoma) [61]. It could be demonstrated that four molecular subtypes, namely POLE mutant (POLEmut), mismatch repair deficient (MMRd), p53 abnormal (p53abn) and no specific molecular profile (NSMP), are independent factors for outcome and survival and could provide guidance in the future for individual treatment like fertility-sparing. The common goal in cancer treatment is to avoid unnecessary side effects and improve the prognosis. The PORTEC4 study has taken an important first step for the treatment of EC.

## Figures and Tables

**Table 1:** Upcoming ESGO and ESTRO recommendations on risk stratification after genomic classification (right) compared to clinical risk stratification (left).

Risk Group	Molecular Classification Un-Known	Molecular Classification Known
**Low**	Stage IA Endometrioid + grade 1–2 + LVSI negative	Stage I–III POLE mutant Stage IA Endometrioid + grade 1–2 + LVSI negative
**Intermediate**	Stage IB Endometrioid + grade 1–2 + LVSI negative Stage IA Endometrioid + grade 3 + LVSI negative	Stage IB Endometrioid + grade 1–2 + LVSI negative MMRd or NSMP Stage IA Endometrioid + grade 3 + LVSI negative MMRd or NSMP
**High-Intermediate**	Stage I Endometrioid + substanial LVSI, regardless of grade and depth of invasion Stage IB Endometrioid grade 3, regardless of LVSI status Stage II	Stage I Endometrioid + substantial LVSI, regardless of grade and depth of invasion, MMRd or NSMP Stage IB Endometrioid grade 3, regardless of LVSI status, MMRd or NSMP Stage II, MMRd or NSMP
**High**	Stage III with no residual disease Non endometrioid (serous, clear cell, undifferentiated carcinoma, carcinosarcoma, mixed)	p53abn regardless of type or stage Stage III Endometrioid with no residual disease, MMRd or NSMP Non endometrioid (serous, clear cell, undifferentiated carcinoma, carcinosarcoma, mixed)
**Advanced** **Metastatic**	Stage III with residual disease & IVA Stage IVB	Stage III with residual disease & IVA Stage IVB

**Table 2:** Suggestions for future recommendations for adjuvant treatment according to the new risk stratification.

New Risk Groups According to Figure 1	Future Adjuvant Treatment Recommendations (Suggestions)		
	Brachytherapy	External Beam Radiation	Chemotherapy
Low Risk	no	no	no
Intermediate Risk	yes	no	no
High-Intermediate Risk pN0 (surgical staging or SLN neg.)	yes	If substantial LVSI	no
High-Intermediate Risk cN0/pNX (no surgical staging nor SLN)	yes	If substantial LVSI	Especially for NSMP grade 3 (see PORTEC3 protocol)
High Risk p53+ regardless of stage; stage IIIC1 NSMP/MMRd, no residual tumor	no	Concurrent chemoradiation +chemo (see PORTEC3 protocol) In case of contra-indications against chemo: EBRT recommended	
High Risk stage III C2	no	Extended field radiation: Concurrent chemoradiation + chemo (see PORTEC3 protocol) In case of contra-indications against chemo: EBRT recommended	
